# Acute Liver Failure Secondary to Primary Large B-cell Lymphoma Complicated by Malignancy-related Hemophagocytic Syndrome: A Case Report

**DOI:** 10.7759/cureus.87288

**Published:** 2025-07-04

**Authors:** Alejandro Rivera Tapia, Mariana De La Torre, Cesar A Nieves Perez, Miguel C Molina Obana, Sarah Diaz Gonzalez

**Affiliations:** 1 General Medicine, Universidad del Valle de Mexico, Hermosillo, MEX; 2 Medicine, Universidad Autónoma de Guadalajara, Zapopan, MEX; 3 Internal Medicine, Hospital Angeles Pedregal, Mexico City, MEX

**Keywords:** acute liver failure (alf), diffuse large b cell lymphoma (dlbcl), hemophagocytic syndrome (hs), lymphoma, non-hodgkin lymphoma (nhl)

## Abstract

Acute liver failure is a catastrophic condition characterized by the sudden and severe impairment of hepatic function. We report the case of a 36-year-old patient who presented with significant dehydration, generalized jaundice, evidence of hepatic encephalopathy, generalized edema with abdominal distension, and hepatosplenomegaly. A liver biopsy was performed, yielding the final diagnosis of primary large B-cell lymphoma. Subsequently, a bone marrow aspirate and biopsy were performed, confirming the diagnosis of hemophagocytic syndrome. The patient was managed with R-CHOEP (rituximab, cyclophosphamide, doxorubicin, vincristine, etoposide, prednisone) chemotherapy. After 21 days, the patient was successfully discharged to continue treatment on an outpatient basis.

## Introduction

Acute liver failure (ALF) refers to the development of severe hepatic dysfunction characterized by an international normalized ratio (INR) >1.5 and altered mental status due to hepatic encephalopathy in a patient without pre-existing liver disease and with a disease duration of <26 weeks [1].

Non-Hodgkin lymphomas (NHLs) rank as the fifth to ninth most common cancers worldwide. Diffuse large B-cell lymphoma (DLBCL), is the most frequently reported, accounting for approximately 40% of NHL cases in North and South America [2]. Liver malignancies are classified as primary or secondary: primary tumors originate within the liver, while secondary tumors result from the dissemination of neoplasms from other primary sites [3]. Primary large B-cell lymphoma of the liver is rare, representing only 1% of extranodal NHL cases. Generally, NHL may cause liver dysfunction in 16-43% of cases due to secondary tumor infiltration at advanced stages, but it is rarely associated with ALF [4]. Only 0.44% of ALF cases are attributed to hematological causes [5]. Since primary large B-cell lymphomas represent 1% of patients and ALF of hematological origin are only 0.44%, this entity can be considered extremely rare [4,5].

## Case presentation

A 36-year-old male from León, Guanajuato, Mexico, presented with symptoms of an upper respiratory tract infection, which began two weeks prior, associated with close contact with one of his children who had similar symptoms. He consulted a private physician who prescribed non-specified non-steroidal anti-inflammatory drugs (NSAIDs). Subsequently, the patient developed progressive asthenia, adynamia, general malaise, abdominal pain, and generalized jaundice. At the private clinic, initial laboratory investigations revealed significantly elevated transaminases, hyperbilirubinemia, cholestatic enzyme elevations, coagulopathy, renal dysfunction, hyponatremia, and pancytopenia. His medical history was significant for a previous episode of hepatitis A one year prior, with no complications. He denied alcohol, tobacco, or drug use, high-risk sexual behavior, or other significant medical history. His laboratory parameters are summarized in Table 1.

**Table 1:** Initial Laboratory Results on Presentation Laboratory results obtained at presentation demonstrated markedly elevated transaminases (AST and ALT), cholestatic enzymes (GGT and ALP), and lactate dehydrogenase. Total and direct bilirubin levels were significantly increased. Coagulation studies revealed prolonged PT, aPTT, and elevated INR. The patient also exhibited hyponatremia, hyperkalemia, azotemia, and elevated serum creatinine. Hematologic parameters showed leukopenia with relative neutrophilia, lymphopenia, thrombocytopenia, and normocytic anemia.

Parameter	Abbreviation	Patient Value	Reference Range
Aspartate aminotransferase	AST	1443 U/L	10–34 U/L
Alanine aminotransferase	ALT	748 U/L	7–55 U/L
Gamma-glutamyl transferase	GGT	385 U/L	12–64 U/L
Alkaline phosphatase	ALP	366 U/L	40–150 U/L
Lactate dehydrogenase	LDH	2613 U/L	125–243 U/L
Amylase	—	44.5 U/L	25–125 U/L
Lipase	—	86 U/L	8–78 U/L
Total bilirubin	TBil	25.124 mg/dL	0.2–1.2 mg/dL
Direct bilirubin	DBil	21.488 mg/dL	0.0–0.5 mg/dL
Indirect bilirubin	IBil	3.636 mg/dL	0.0–1.0 mg/dL
Blood urea nitrogen	BUN	46.6 mg/dL	8.9–20.6 mg/dL
Serum creatinine	Cr	2.48 mg/dL	0.7–1.3 mg/dL
Serum sodium	Na⁺	121.9 mEq/L	136–145 mEq/L
Serum potassium	K⁺	5.6 mEq/L	3.5–5.1 mEq/L
Prothrombin time	PT	26.2 seconds	8.8–13.4 seconds
Activated partial thromboplastin time	aPTT	44 seconds	25–45 seconds
International normalized ratio	INR	2.37	0.8–1.2
Hemoglobin	Hb	13.3 g/dL	14–18 g/dL (men)
Hematocrit	Hct	38%	42–54% (men)
Platelet count	Plt	118.4 × 10³	130–400 × 10³/µL
White blood cell count	WBC	3.82 × 10³/µL	3.8–11.2 × 10³/µL
Lymphocyte percentage	Lym%	0.34%	20–40%
Neutrophil percentage	Neu%	82%	40–70%

Despite treatment, the patient showed no improvement and developed healthcare-associated pneumonia, prompting transfer to our facility for medical management and further evaluation. Upon arrival at the emergency department, the patient appeared dehydrated, with generalized jaundice, signs of hepatic encephalopathy, generalized edema, abdominal distension, and hepatosplenomegaly. Initial imaging (non-contrast chest and cranial computer tomography (CT)) revealed interstitial pneumonia in the right lower lobe with a 25% pleural effusion; the cranial CT was unremarkable. Treatment was initiated with weight-based acetylcysteine, lactulose, and rifaximin.

On the second day of hospitalization, high-flow nasal cannula support was started, without vasopressor requirement. Due to hypofibrinogenemia, recombinant fibrinogen concentrate was administered. Ampicillin and valganciclovir were discontinued, and meropenem was initiated. A transjugular liver biopsy was performed. On the third day, due to inadequate clearance and acute kidney injury (AKI) stage III, a Mahurkar catheter was placed for acute hemodialysis, along with hypertonic saline for hyponatremia and 50% dextrose infusion for recurrent hypoglycemia. On the fourth day, polymerase chain reaction (PCR) confirmed pneumonia caused by *Haemophilus influenzae*, prompting the replacement of meropenem with cefotaxime.

**Figure 1:**
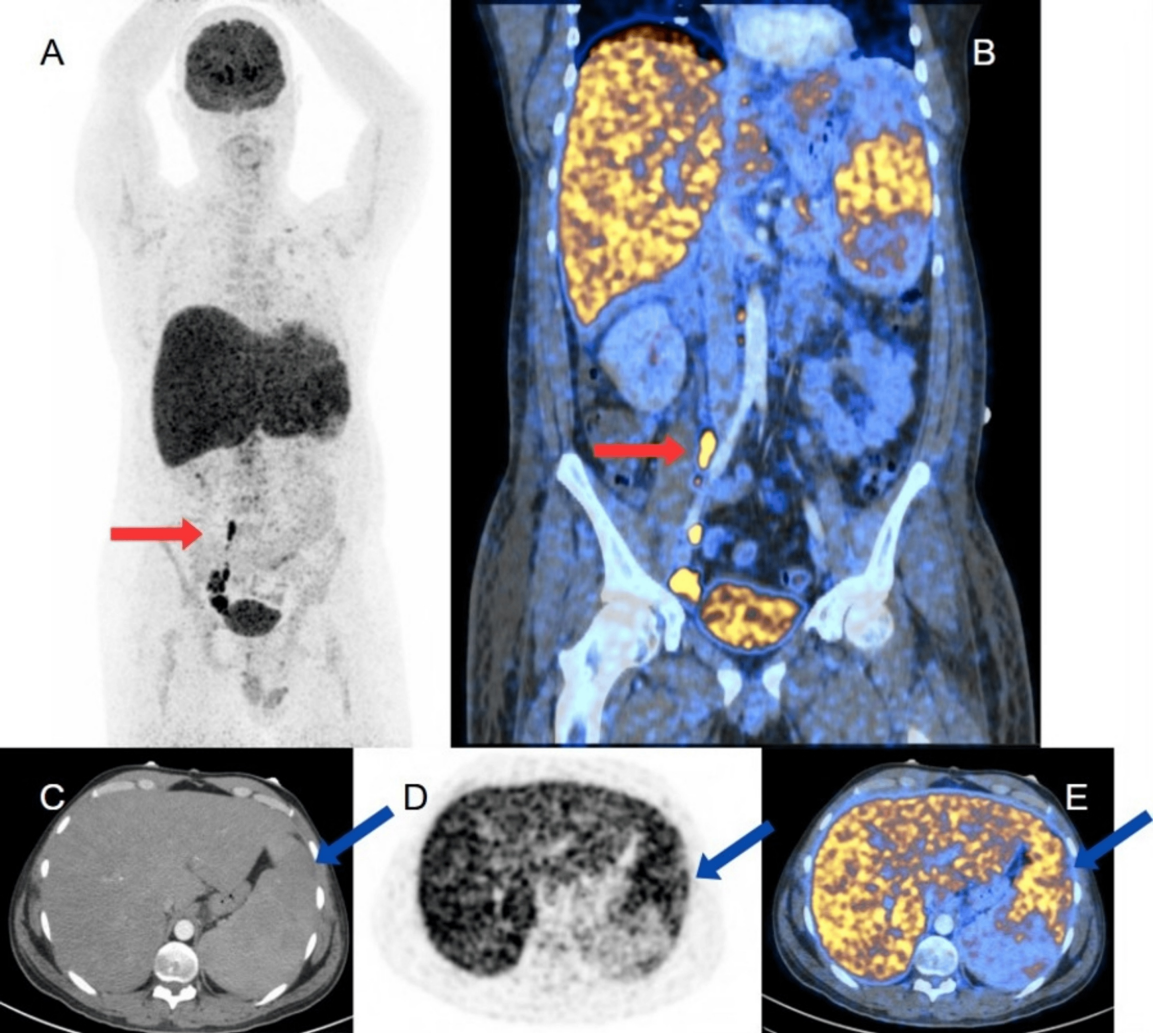
18F-FDG PET-CT images (A) Coronal PET and (B) Coronal PET/CT fusion show increased focal metabolic activity in the spleen and abdominopelvic lymph nodes (red arrows), and diffuse metabolic activity in the liver, associated with hepatosplenomegaly. (C) Axial CT in arterial phase, (D) Axial PET, and (E) Axial Lumbar Interbody Fusion demonstrate a heterogeneous spleen with multiple lesions (blue arrow) and a liver with diffuse metabolic increase. 18F-FDG: 2-deoxy-2-[^fluorine-18^]fluoro-D-glucose

On the fifth day, dexamethasone was initiated due to suspected hemophagocytic lymphohistiocytosis (HLH) (score: 201), characterized by fever, hepatosplenomegaly, hypofibrinogenemia, hypertriglyceridemia, and reduced natural killer (NK) cells. Positron emission tomography (PET)/CT revealed increased focal metabolic activity in the spleen, abdominopelvic lymph nodes, and diffuse liver and bone marrow uptake, suggesting a lymphoproliferative process (Figure 1). 

**Figure 2:**
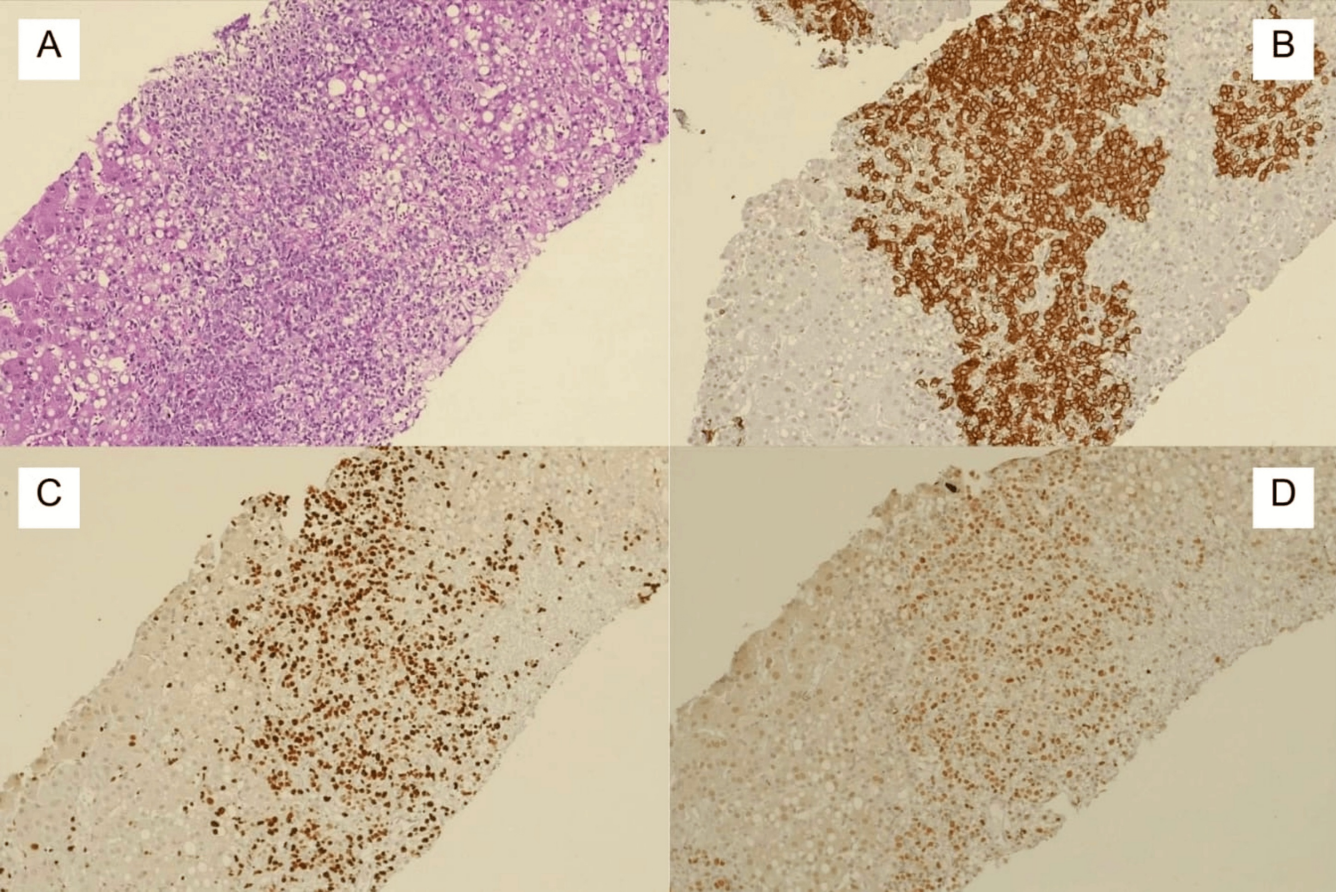
Hepatic infiltration by large B-cell lymphoma: histological and immunohistochemical findings Liver biopsy shows destruction of the hepatic architecture with a pattern of portal and sinusoidal infiltration by large atypical lymphoid cells, characterized by vesicular nuclei, prominent nucleoli, and abundant cytoplasm, features are consistent with large cells (A) (hematoxylin and eosin, 10x). Neoplastic cells are seen to express CD20 (B), CD30, MUM1 (50%), Ki67 (95%) (C), BCL6 (D) (immunohistochemistry, 10x), and BCL2; and are negative for C-MYC, CD3, and CD5.

On the seventh day, liver biopsy (Figure 2) confirmed large B-cell NHL with portal and sinusoidal infiltration. Immunophenotype included CD20+, CD30+, MUM1+, BCL6+, BCL2+, C-MYC-, CD3-, CD5-, CD21-, CD56-, CK7-, Ki-67: 95%, Epstein-Barr virus (EBV)-encoded RNA (EBER) (-).

**Figure 3:**
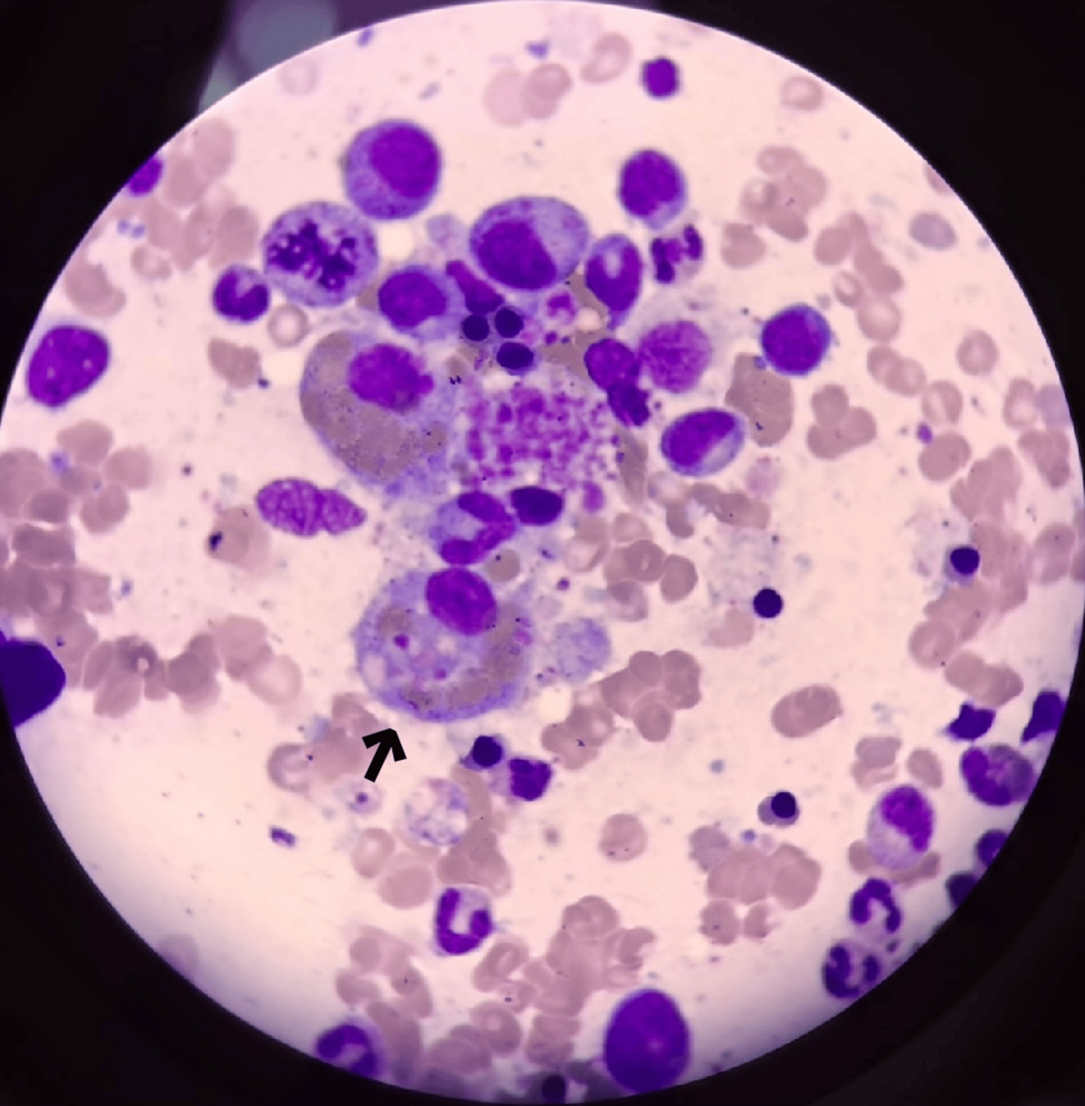
Bone marrow smear (Wright-Giemsa stain) showing features of hemophagocytosis lymphohistiocytosis A large macrophage at the center (arrow) with abundant cytoplasm is observed, containing numerous phagocytosed hematopoietic cells in various stages of degradation, including erythrocytes and myeloid/erythroid precursors.

Bone marrow biopsy (Figure 3) confirmed hemophagocytosis on the ninth day, leading to systemic dexamethasone initiation. On the tenth day, R-CHOEP (rituximab, cyclophosphamide, doxorubicin, vincristine, etoposide, prednisone) chemotherapy was started, adjusted for renal function.

During hospitalization, the patient received three R-CHOEP cycles. He developed severe febrile neutropenia complicated by mucositis (grade III) and nosocomial pneumonia due to *Burkholderia cepacia*. The patient was hospitalized for 31 days, showing clinical and laboratory improvement. After six hemodialysis sessions, renal function was restored to normal levels. The Mahurkar catheter was removed at discharge, and the patient continued with outpatient chemotherapy.

## Discussion

Although NHL is a common lymphoproliferative entity, hepatic involvement is rare, occurring in only 10% of patients, with primary hepatic presentation in less than 1% [5]. This atypical presentation makes diagnosis challenging and is associated with high mortality, estimated at approximately 90% within 6-12 days from symptom onset, highlighting the importance of a thorough and systematic diagnostic approach [6].

ALF is a heterogeneous condition with multiple differential diagnoses, requiring extensive clinical history and diagnostic evaluation. In this case, the complexity is underscored by the fact that DLBCL accounts for only 0.26% of ALF cases [7]. However, it is essential to consider DLBCL as a differential diagnosis in ALF. In a case reported by Shibata et al., a 33-year-old patient presented with ALF as the initial manifestation of DLBCL despite the absence of lymphadenopathy or specific imaging findings [4]. Due to coagulopathy, a liver biopsy could not be performed, but a gastric biopsy confirmed the diagnosis, allowing initiation of rituximab therapy and subsequent clinical resolution. Other authors emphasize the need to include DLBCL in the diagnostic workup for unexplained ALF, especially in abnormal liver function tests and disproportionately elevated lactate dehydrogenase (LDH) without an apparent cause [6].

The treatment of this condition is not standardized and varies depending on the severity of the clinical presentation. Orthotopic liver transplantation (OLT) has been used in selected cases of fulminant hepatic failure secondary to primary hepatic lymphoma. One documented case describes a patient who underwent successful OLT followed by R-CHOP chemotherapy, resulting in excellent clinical recovery [8]. However, current guidelines do not include OLT as a formal recommendation in these scenarios due to limited evidence and the potential risk of tumor recurrence. This decision remains individualized and context-dependent, with chemotherapy as the primary therapeutic approach in most cases [9,10].

From a histopathological perspective, the patient’s immunophenotypic profile, positive for CD20, CD30, BCL6, MUM1, and BCL2, with a high proliferation index (Ki-67: 95%), is indicative of an activated B-cell subtype of DLBCL. This subtype, characterized by the absence of CD10 and the presence of MUM1, has been associated with poorer prognosis and reduced response to R-CHOP therapy compared to the germinal center subtype [11,12]. Furthermore, CD30 expression, although uncommon, may be observed in some aggressive DLBCL variants, including EBV-positive forms [10]. In this context, the absence of EBER rules out the EBV-positive subtype, which is often associated with a worse prognosis, particularly in older adults. Such patients typically exhibit lower response rates to R-CHOP, driving the exploration of targeted therapeutic strategies such as brentuximab vedotin [10-12].

Given the aggressive clinical presentation, histopathological findings indicating an activated B-cell (ABC) subtype of DLBCL, and concurrent hemophagocytic syndrome, the R‑CHOEP regimen was selected over standard R‑CHOP. Although our patient tested negative for EBER, the presence of CD30 and the high proliferation index (Ki-67: 95%) supported a more aggressive disease course. Etoposide was added to enhance cytotoxic activity against both lymphoma cells and hyperactivated macrophages involved in HLH. This approach is supported by recent evidence showing improved clinical outcomes in adult HLH with etoposide-containing regimens [13-14].

## Conclusions

This case highlights a rare presentation of ALF due to primary hepatic DLBCL, a condition with high mortality and diagnostic challenges. Despite its rarity, DLBCL should be considered in patients with unexplained ALF, mainly when elevated liver enzymes and disproportionate LDH are observed. Early recognition and prompt initiation of targeted therapy, such as R-CHOP or even liver transplant should be considered. While standard treatment with R-CHOP was adequate, the immunophenotype (CD20+, CD30+, MUM1+, BCL6+, BCL2+) suggested an aggressive subtype, highlighting the potential role of targeted therapies, such as brentuximab vedotin, for improved outcomes. Further research is needed to refine diagnostic approaches and optimize management strategies for this rare presentation.
